# A case of human trombiculosis caused by *Eutrombicula alfreddugesi* (Acariformes: Prostigmata: Trombiculidae) in Chamela, Jalisco, Mexico

**DOI:** 10.1093/jme/tjag156

**Published:** 2026-10-01

**Authors:** Ricardo Paredes-León, Carmen Guzmán-Cornejo

**Affiliations:** Colección Nacional de Ácaros, Instituto de Biología, Universidad Nacional Autónoma de México (UNAM), Ciudad de México, México; Laboratorio de Acarología, “Anita Hoffmann”, Facultad de Ciencias, Universidad Nacional Autónoma de México (UNAM), Ciudad de México, México

**Keywords:** chigger, tlalzahuatl, Estación de Biología Chamela, tlalzahuatiasis

## Abstract

A case of trombiculosis is presented at the Estación de Biología Chamela in Jalisco, Mexico. This occurred during an expedition in search of parasites in the fall of 2024. One of the participants presented small red macules on her shins and ankles, and when these were observed with a portable magnifying glass, the presence of chiggers (Trombiculidae) was confirmed. The mites were identified as *Eutrombicula alfreddugesi* (Oudemans), which caused severe trombiculosis due to hypersensitivity in the affected person. In addition to the morphological diagnosis of the chiggers found, a clinical description of the case is presented.

## Introduction

Chiggers (Acariformes: Prostigmata: Trombiculidae) are parasitic mites in the larval instar, whereas subsequent stages are free-living. Their hosts are terrestrial vertebrates (Walter et al. 2009, Mullen and OConnor 2019). More than 3,000 species are recognized worldwide, and some of them are of medical and veterinary importance, either as transmitters of pathogenic microorganisms or as causes of chigger dermatitis or trombiculosis (Nielsen et al. 2021, Moniuszko et al. 2022).

*Eutrombicula alfreddugesi* (Oudemans) is the most common and widespread chigger species in America, distributed from Canada to Argentina and the West Indies (Wharton and Fuller 1952, Hoffmann 1990, Lareschi 2004). Although some authors consider *E. cinnabaris* as the most common chigger in the United States, a species that was long considered a synonym of *E. alfreddugesi* (Bennett et al. 2014, Chen et al. 2022). In Mexico,*E. alfreddugesi* is the most common chigger occurring in all the states, parasitizing around 80 host species, including humans (Hoffmann 1990). The discomfort caused by this chigger was known since pre-Hispanic times, and the ancient Mexicans referred to them as “tlalzahuatl,” which in the Nahuatl language means “scabies that comes from the soil” (Hoffmann 2007). The name tlalzahuatl was used by Altamirano (1891) and Dugès (1891) to refer to *E. alfreddugesi*; however, these early and subsequent records of trombiculosis in Mexico (e.g. Islas 1943, Bada-del Moral et al. 2015, Ayala-Cortés et al. 2016) do not allow us to determine with certainty the specific identity of the causative agent of these dermatoses.

Trombiculosis (also known as tlalzahuatiasis) in Mexico is an underestimated disease without morbidity statistics. To the best of our knowledge, there are no confirmed cases of trombiculosis caused by *E. alfreddugesi* in Mexico. By confirmed cases, we mean cases where mites were recovered directly from the skin lesions and taxonomically determined. In this context, this contribution reports a case of human trombiculosis caused by *E. alfreddugesi* in Chamela, Jalisco, Mexico.

## Materials and Methods

During a school practice in collecting parasitic metazoans at the Estación de Biología Chamela of the Universidad Nacional Autónoma de México (UNAM) in Jalisco, Mexico (19.498734° N, 105.042160° W), one of the participants had some small arthropod bites on her legs. In this region, the predominant vegetation type is the tropical deciduous forest. The collection was conducted during the last month (October 2024) of the rainy season. We toured paths approximately 2 meters wide, and some other narrower secondary paths, where only one person can pass at a time, and often shaded by small trees typical of the dry forest.

The mites were collected directly from the legs using a portable magnifying glass (10×) and entomological needles, and were preserved in a vial with 80% ethanol. Posteriorly, mites were cleared and mounted with Hoyer’s medium between microscope slides and coverslips following the method by Walter and Krantz (2009). The specimens were identified using the keys of Brennan and Goff (1977) and Hoffmann (1990), using a phase and differential interference contrast microscope (Nikon Optiphot-2). Photographs were obtained with a digital camera mounted in an inverted optic microscope (Olympus IX-81).

Mites were collected under the collection permit SPARN/DGVS/13333/24 granted to R. Paredes-León. All the mites are deposited in the Colección Nacional de Ácaros (CNAC) of the Instituto de Biología, UNAM.

## Results and Discussion

### Taxonomy

We collected 7 specimens (CNAC012714 to 012717) from the skin lesions on the legs of the infested person. All of them were identified as larvae of *Eutrombicula alfreddugesi* (Fig. 1A). These specimens fit with the diagnostic characteristics of the species according to the redescription of Bassini-Silva et al. (2024): adoral (*cs*) setae nude (Fig. 1B); number of opisthosomal setae on dorsal rows C, D, E, F and H: 8, 6, 4, 2 and 2, respectively (Fig. 1D); famulus ε on tarsus I distal and ε on tarsus II proximal to solenidion (ω) (Fig. 1F); prodorsal sclerite with a pair of *ve*, *sci* (flagelliform trichobothria), *sce* and one *vi* (Fig. 1E).

**Fig. 1.**
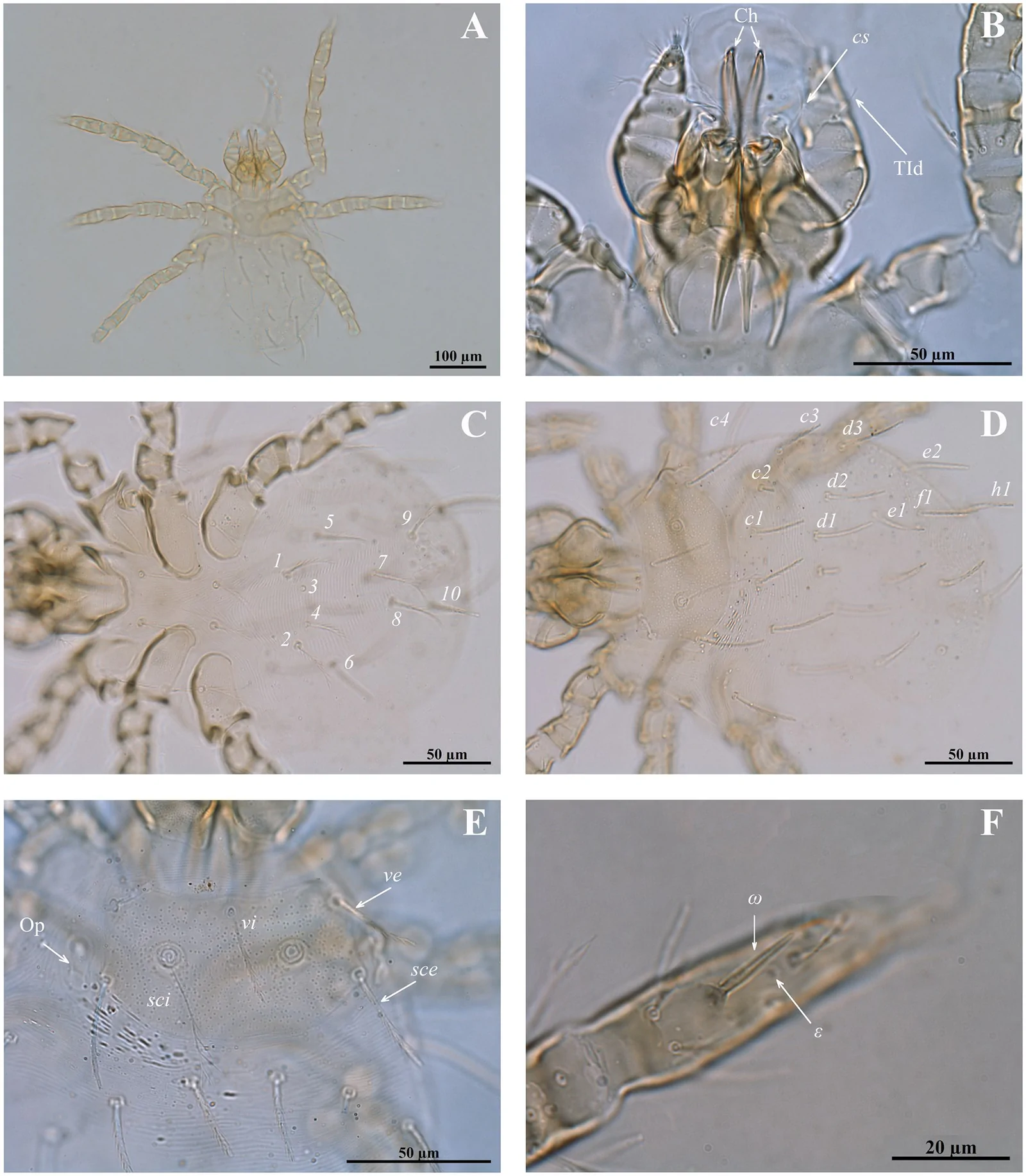
Light microscopy images of *Eutrombicula alfreddugesi* larvae. A) Dorsal view of a complete specimen. B) Dorsal view of gnathosoma. C) Ventral view of idiosoma. D) Dorsal view of idiosoma. E) Prodorsal sclerite. F) Tarsus I. Symbols: Ch = chelicerae; *cs* = adoral setae; TId = dorsal seta of palptibia; numbers 1 to 10 indicate the ventral opisthosomal setae; *c1* to *c4*, *d1* to *d3*, *e1* to *e2*, *f1*, and *h1 *= opisthosomal setae of the C, D, E, F and H dorsal rows, respectively; Op = ocular plate; *sce* = external scapular setae; *sci* = internal scapular setae (flagelliform trichobothria); *ve* = external vertical setae; *vi* = internal vertical seta; *ω* and *ε* = solenidion and famulus on tarsus I, respectively.

However, we observed morphological differences in the collected specimens with respect to some diagnostic characters of the type specimens according to Bassini-Silva et al. (2024):

1. The palpal setal formula was B/N/NNB (specimens CNAC012714 and 012715) (Fig. 1B) or B/B/NNB (specimen CNAC012716) instead of B/B/BNB. In the case of the palpal genu seta, Fuller (1952) had already noticed variation in the specimens now known as lectotype and paralectotype, indicating that in a single specimen one palp was nude (=N) and in the other palp the seta had a single branch (=B) proximal at midpoint.
2. Our specimens have 10 ventral opisthosomal setae instead of 12. The most posterior pair of setae is absent (Fig. 1C).
3. Presence of one nude mastiseta (absent in lectotype and paralectotype) (Bassini-Silva et al. 2024).

Despite the differences observed, we considered our specimens as conspecific with *E. alfreddugesi*, until we have a larger ­sample of specimens that allows us to assess whether it is intra- or inter-specific variation.

### Signs of Hypersensitivity (Allergy) to Mite Bites

The infested person, a 50-yr-old woman, began experiencing the first symptoms approximately 2 h after walking along the narrow trails of the Estación de Biología Chamela (Fig. 2A to C). These consisted of the presence of small red macules on the shins and ankles, with a slight itch. At this time, some of the mites were removed (Fig. 2D and E). In the following 6 to 12 h, the lesions become urticarial papules and itch intensely. The skin with the lesions becomes a pruritic, pale red, flat, and conical lesions. These papules became exceedingly hemorrhagic and deep red (Fig. 2F). Some papules became a vesicle containing clear fluid and the itching was intense (Fig. 2G). The affected person also experienced pain in the areas around the injuries, throughout the leg from the knee down, and swelling in the ankles and feet.

**Fig. 2.**
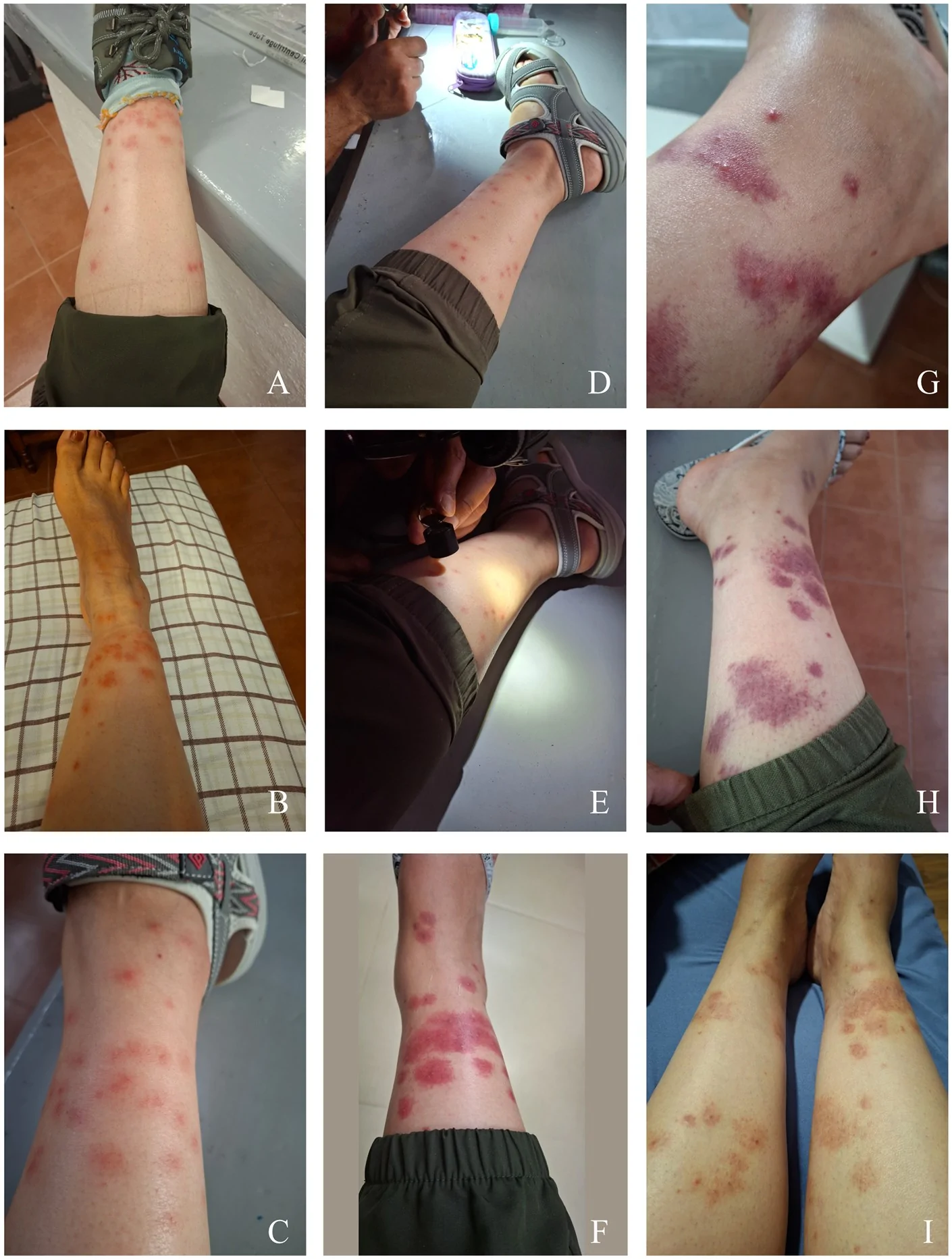
Sequence of symptoms due to contact with chiggers of the species *Eutrombicula alfreddugesi* at the Estación de Biología Chamela, Jalisco, Mexico (October 2024). A to C) Injuries caused by chiggers after fieldwork (21st). D and E) Examination of lesions and collection of chiggers with the aid of a magnifying glass (10×) and entomological needles (same date). F) Red, flat lesions (22nd). G) Some fluid-filled blisters (23rd). H) Purple coloration around blisters (23rd). I) Ciudad de México. Decreased pigmentation of the lesions (28th).

Approximately 24 h after the onset of the first symptoms, the person decided to go to a clinic. After examining the injuries and questioning the patient, the medical doctor diagnosed them as chigger bites with hypersensitivity, and prescribed prednisone (¼ of a 20 mg tablet every 8 h for 3 d) and a single dose of ivermectin (3 tablets of 6 mg). The itch disappeared shortly after the medication, and the skin around the blisters turned purple (Fig. 2H). Gradually, the coloration of the lesions was decreasing over the days (Fig. 2I).

The doctor stated that the excessive reaction to the chiggers was due to previous contact. The affected person reported that in the first days of November 2019, she visited the Estación de Biología Chamela and experienced the same symptoms after returning from fieldwork, but on that occasion, she had a greater formation of fluid-filled blisters. Experimentally, evidence has been provided that continuous introduction of chigger saliva into mice generates Arthus-type hypersensitivity responses (Wright et al. 1988). The Arthus response is a Type III Hypersensitivity Reaction, in which, if an animal or human subject is injected intradermally with an antigen to which large amounts of circulating antibodies exist, antigen will diffuse into the walls of local blood vessels and large immune complexes will precipitate close to the injection site. This initiates an inflammatory reaction. Inflammation at the site of an Arthus reaction is characterized by swelling and localized bleeding, followed by fibrin deposition (Delves et al. 2017, Punt et al. 2019, Male et al. 2026).

The Estación de Biología Chamela, UNAM, is a highly visited research center, attracting not only national but also international researchers; in 2025 alone, 523 visits were recorded (Renton pers. comm.). All these visitors may be exposed to varying degrees to the bites of chiggers, and although not everyone experiences hypersensitivity, the damage caused by these cases of trombiculosis represents a potential risk.

Accurate identification of the causative agents of these trombiculosis cases is crucial for implementing measures to reduce exposure to them and to have, as far as possible, a timely diagnosis and treatment to reduce the symptoms. Systematic data collection will help determine whether the morphological variations detected in the Chamela specimens represent intra- or interspecific variation. To date, no pathogenic microorganisms have been detected in *Eutrombicula alfreddugesi* (Moniuszko et al. 2022), but further research is needed on this important public health issue.
